# *Cyberlindnera jadinii* yeast as a functional protein source: Modulation of immunoregulatory pathways in the intestinal proteome of zebrafish (*Danio rerio*)

**DOI:** 10.1016/j.heliyon.2024.e26547

**Published:** 2024-02-21

**Authors:** Kathiresan Purushothaman, Alexander D. Crawford, Sérgio D.C. Rocha, Aleksandar B. Göksu, Byron Morales Lange, Liv Torunn Mydland, Shubha Vij, Lin Qingsong, Margareth Øverland, Charles McL. Press

**Affiliations:** a Department of Preclinical Sciences and Pathology, Faculty of Veterinary Medicine, Norwegian University of Life Sciences, Ås, Norway; b Department of Animal and Aquaculture Sciences, Faculty of Biosciences, Norwegian University of Life Sciences, P.O. Box 5003, Ås, Norway; c School of Applied Science, Republic Polytechnic, 9 Woodlands Avenue 9, Singapore 738964, Singapore; d Tropical Futures Institute, James Cook University Singapore, 149 Sims Drive, 387380, Singapore; e Department of Biological Sciences, National University of Singapore, 14 Science Drive 4, Singapore 117543, Singapore

**Keywords:** Non saccharomyces yeast, Innate immunity, KEGG pathways analysis, iTRAQ, 2D LC-MS/MS, Proteomics

## Abstract

Yeasts contain bioactive components that can enhance fish immune robustness and disease resistance. Our study focused on analyzing intestinal immunoregulatory pathways in zebrafish (*Danio rerio*) using iTRAQ and 2D LC-MS/MS to quantify intestinal proteins. Zebrafish were fed either control diet (C) or diet supplemented with autolyzed *Cyberlindnera jadinii* (ACJ). KEGG analysis revealed that ACJ yeast diet induced increased abundance of proteins related to arginine and proline metabolism, phagosome, C-lectin receptor signaling, ribosome and PPAR signaling pathways, which can modulate and enhance innate immune responses. ACJ yeast diet also showed decreased abundance of proteins associated with inflammatory pathways, including apoptosis, necroptosis and ferroptosis. These findings indicate boosted innate immune response and control of inflammation-related pathways in zebrafish intestine. Our findings in the well annotated proteome of zebrafish enabled a detailed investigation of intestinal responses and provide insight into health-beneficial effects of yeast species *C*. *jadinii*, which is relevant for aquaculture species.

## Highlights

• Bioactive components in a diet containing *Cyberlindnera jadinii* yeast modulate innate immunity-related proteins in the zebrafish intestine.
• Novel feed containing *C. jadinii* yeast reduced inflammation-related proteins of the apoptosis, necroptosis and ferroptosis pathways.
• Increased abundance of proteins related to, phagosome, C-lectin receptor and PPAR signaling pathway are associated with alleviation of inflammation.

## Graphical abstract

**Figure:**
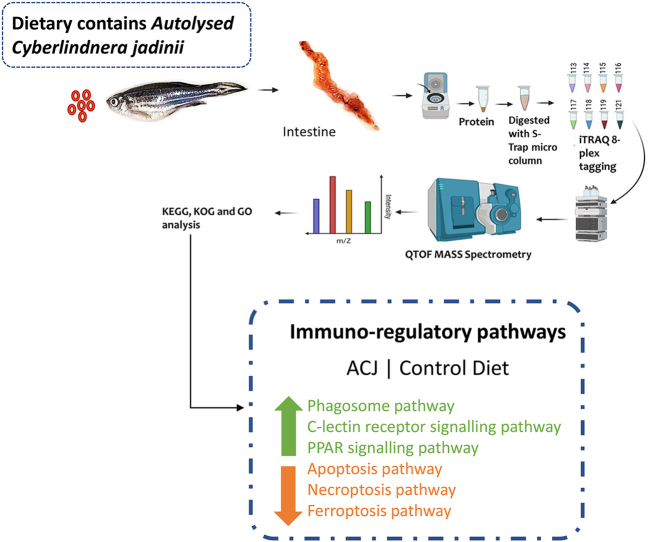

## 1 Introduction

Yeasts are an alternative high-quality protein sources for aquaculture [[1], [2], [3], [4], [5], [6]]. The ability of yeasts to utilize non-food biomass such as wood hydrolysates and chicken by-products as feedstocks makes yeasts a local low environmental-impact ingredient that can contribute to sustainability of aquaculture feed production [7].

In addition, yeasts is a functional protein source that contains health-beneficial bioactive compounds that can help solve challenges associated with high mortalities and morbidities due to multi-stressor factors during aquaculture production such as environmental challenges, stress associated with handling, seawater transfer and exposure to pathogens [8]. Yeasts contain about 50–58% crude protein and bioactive components such as β-glucan, mannoproteins, and nucleic acids that can have positive effects on fish development and immune defense and can contribute to increased resilience [[9], [10], [11], [12]]. However, the health effects depend on the yeast strain, its processing condition and dietary inclusion levels [4,11,13,14]. At laboratory scale, small batches of yeasts are produced and processed which are suited for assessment in small laboratory fish species such as zebrafish (*Danio rerio*) as a pre-screen model to guide studies in aquaculture species.

Zebrafish is a laboratory fish species widely used to model developmental, physiological and pathological conditions in biology. Zebrafish are also increasingly recognized as a model suited to investigation of how diets influences host-microbe-immune interactions and health in fish [15]. The genome of zebrafish is well-annotated, and a diverse set of research tools is available making zebrafish a model organism to investigate immune processes and underlying molecular mechanisms [16,17]. While transcriptomics has been a robust tool for study of immune system in fish, proteomic studies provide a comprehensive survey of the phenotypic profile of the tissue, organ or organism. The proteome is the complete set of proteins expressed by an organism or specific tissue. Proteins are the main components of the physiological and metabolic pathways, and the establishment of protein-protein interaction networks and proteome profiling allows the identification of new biomarkers, disease related pathways and pathological processes. Changes in blood plasma proteome have been investigated in Atlantic salmon (*Salmo salar*) [13] and zebrafish [18], but data on changes in the proteome of intestinal tissues following dietary challenge are scarce. Kiron, Kathiresan [19] investigated alterations in the intestine mucus proteome induced by a functional diet containing a feed additive (nucleotides, antioxidant, vitamins C and E, beta-glucan) in Atlantic Salmon. These investigators found that the functional diet alleviated inflammation and contributed baseline information on the intestinal proteome in Atlantic salmon.

A recent transcriptomic study from our group reported that the yeast *Cyberlindnera jadinii* had health-beneficial effects reducing inflammation associated with feeding plant-based diets to Atlantic salmon [4,20]. It has been reported that *C. jadinii* yeasts controlled inflammatory responses of fish through up-regulation of pathways associated with wound healing processes and modulation of innate immunity through taurine and hypotaurine pathways. Moreover*, C. jadinii* can modulate the protein level of cytokines such as Tumor necrosis factor α and interleukin 10, both classic biomarkers of the balance between pro- and anti-inflammatory responses. To address whether yeast-based diets induce comparable health-beneficial changes in the intestine at the protein level, a study of the intestinal proteome of zebrafish fed the *C. jadinii* yeast containing diet was performed. Most dietary formulations for zebrafish are based on requirements determined in other cyprinids with estimates for protein requirements ranging from 30 to 53% [17]. Reproductive output (fecundity) is an important outcome for zebrafish culture and commercial diets usually contain about 60% protein. On the other hand, diets for Atlantic salmon conventionally contain lower protein levels of about 40–45%. We have already demonstrated that *C. jadinii* yeast-based diet containing 40% protein had health-beneficial effects in salmon.

In the present study, a pilot study was first performed to compare a high protein (“commercial-like”) control diet with a low protein control diet and a low protein test diet with autolysed *C*. *jadinii* yeast (LPY) to demonstrate that the health of zebrafish was not adversely affected by the low protein content. Subsequently, we performed a feeding trial using five diets: a high-protein control diet (C), and four low-protein diets containing 10% of four different yeasts, including autolysed *C*. *jadinii* yeast (ACJ) to demonstrate that zebrafish were not adversely affected by the yeast strain or the down-stream processing method. The autolysed *C*. *jadinii* yeast had been shown by Abgoola et al. (2022) to have health-beneficial effects in Atlantic salmon. Based on the results from the previous studies, intestinal proteome analysis was performed on samples from zebrafish fed the control and the ACJ diet to delineate molecular mechanisms and pathways associated with the immunoregulatory ability of the yeast-based diet.

## 2 Materials and methods

### 2.1 Ethics statement for animal handling

The use of animals was approved by the Norwegian Food Safety Authority (FOTS ID 20998). The approval was granted in accordance with Norwegian regulations of June 18, 2015 No 761 concerning the use of animals for scientific purposes (Regulations) § 37, cf. § 6.

### 2.2 Fish husbandry and feeding strategy

The adult zebrafish (AB strain) were reared following well established protocols (ref. zfin.org & zebrafish book). The zebrafish were raised in ZebTec Active Blue recirculating systems (Tecniplast, Buguggiate, Italy) at an ambient temperature (26–28 °C), pH (7.0–7.5), and photoperiod of 14/10 h light/dark cycle. The embryos (400–500) were produced from a mass crossing. Starting five days-post-fertilisation (dpf) the larvae were fed with zebrafeed (Zebrafeed, Sparos Ltd., Olhão, Portugal) size <100, followed by artemia (Sep-Art Artemia, Ocean Nutrition, Belgium) and zebrafeed 100–200 beginning at 14 dpf, and then advanced to feeding with zebrafeed 200–400 μm at 30 dpf. Two months-post-fertilisation (mpf), zebrafish of uniform size were stocked in groups of 45 in 9 L recirculating tanks and fed zebrafeed 400–600, supplemented with artemia.

### 2.3 Diets

**For the pilot study, three experimental diets** were produced at Sparos Ltd. The three diets were a high protein diet (HP) with ca. 63% protein, a low protein diet (LP) with ca. 42% protein and a low protein diet with ca. 42% protein that included autolyzed *C. jadinii* (LPY) (Supplementary File S1).

**For the feeding trial, five experimental fish diets** were produced at the Centre for Feed Technology, Norwegian University of Life Sciences, Ås, Norway. These were a control diet (C) contained ca. 60% crude protein, and four experimental diets containing 44% crude protein and 10% of one of four yeast ingredients, which were *C. jadinii* (intact: ICJ or autolyzed: ACJ) or *Wickerhamomyces anomalus* yeast (intact: IWA or autolyzed: AWA). Detailed information about yeast processing is reported elsewhere [4,11]. The feed formulation and composition of the five diets used in the feeding trail are presented in Table 1, Table 2. Briefly, dry ingredients were thoroughly mixed and only then liquid components such as water, gelatin and fish oil were added. The mash was cold pelleted into 0.5 mm diameter by using P35A pasta extruder (Italgi, Italy). The pellets were dried at approximately 55 °C until <10% moist and stored at 4 °C prior to feeding. Chemical analysis of the diet composition (ground at 0.5 mm) was performed in duplicates by the LabTek group at the Department of Animal and Aquacultural Sciences, NMBU (Table 2). Dry matter, ash, crude protein and crude lipid were analyzed according to the methods described in the European Commission Regulation No 152/2009 [21]. Starch was hydrolyzed with α-amylase and amyl glucosidase-enzymes to glucose, and the glucose concentration was determined spectrophotometrically (RX4041 Randox Daytona+, Randox Laboratories, Antrim, UK) as described by McCleary, Solah and Gibson [22]. Gross energy content was determined using a PARR 6400 Automatic Isoperibol Calorimeter (Parr Instruments, Moline, Illinois, USA) according to ISO 9831 [23]. Total phosphorous content was measured using a microwave plasma atomic emission spectrometer (MP-AES 4200, Agilent Technologies, USA) after combustion and acid decomposition in a Start D microwave digestion system (Milestone Srl, Italy).

**Table 1:** Composition of diets used in feeding trial.

					
Ingredients (g kg^−1^)	C	ICJ	ACJ	IWA	AWA
Fish meal^a^	440	200	200	200	200
Soybean protein concentrate^b^	93	163	163	163	163
Soybean meal^c^	60	80	80	80	80
Wheat gluten meal^d^	90	70	70	70	70
Gelatinized potato starch^e^	120	170	170	170	170
Fish oil^f^	110	130	130	130	130
Monocalcium^g^	15	15	15	15	15
vitamin and mineral premix^h^	5	5	5	5	5
Gelatin^i^		60	60	60	60
L-lysine^j^	4	4	4	4	4
DL-Methionine^k^	1.5	1.5	1.5	1.5	1.5
Choline chloride^l^	1.5	1.5	1.5	1.5	1.5
ICJ^m^	0	100	0	0	0
ACJ^m^	0	0	100	0	0
IWA^m^	0	0	0	100	0
AWA^m^	0	0	0	0	100

a Fish Powder SKPT, 0268, SeaGarden, Norway.

b Soybean protein concentrate, Tradkon SPC HC-200, Sojaprotein, Becej, Serbia.

c Soybean meal, Denofa AS, Fredrikstad, Norway.

d Wheat gluten, Amilina AB, Panevezys, Lithuania.

e Lygel F 60, Lyckeby Culinar, Fjälkinge, Sweden.

f NorSalmOil, Norsildmel, Egersund, Norway.

g Monocalcium phosphate, Bolifor MCP-F, Oslo, Norway Yara.

h Vit/min premix, NMBU Fish Premix Basic, Trouw Nutrition, LA Putten, The Netherlands. Per kg of feed**:** Vitamin A 2500 IU; Vitamin D2 1500 IU; Vitamin E (all-rac-alpha-tocopheryl acetate) 200 IU; Vitamin K3 (Menadione nicotinamide bisulfite) 10.05 mg; Vitamin B1 (Thiamine mononitrate) 15.0 mg; Vitamin B2 (Riboflavin) 25.0 mg; Calcium D-pantothenate 40.0 mg; Niacinamide 75.05 mg; Vitamine B6 (pyridoxine hydrochloride) 15.0 mg; Folic acid 5.0 mg; Vitamin B12 (cyanocobalamin) 25.0 mg; Vitamin C 125.0 mg; Biotin 275.0 mg; Calcium iodate, anhydrous, Iodine 30.0 mg; Manganese (II) oxide, Manganese 15.0 mg; Zinc oxide, Zinc 105.0 mg. Carrier: Calcium carbonate.

i Rousselot 250 PS, Rousselot SAS, Courbevoie, France.

j L-Lysine CJ Biotech CO., Shenyang, China.

k Rhodimet NP99, Adisseo ASA, Antony, France.

l Choline chloride, 70% Vegetable, Indukern SA., Spain.

m Agboola et al., 2022 (Inter. Jour. Mol. Sci.).

**Table 2:** Analyzed chemical composition.

	C	ICJ	ACJ	IWA	AWA
*Dry matter (%)*	92.8 ± 0.01	92.1 ± 0.04	92.2 ± 0.07	92.3 ± 0.13	92.9 ± 0.05
*Crude protein (%)*	60.2 ± 0.15	47.7 ± 0.22	46.8 ± 0.06	47.6 ± 0.05	47.8 ± 0.40
Crude lipid (%)	10.5 ± 0.03	12.4 ± 0.11	14.2 ± 0.02	12.9 ± 0.08	12.9 ± 0.02
Ash (%)	5.22 ± 0.04	4.83 ± 0.05	4.70 ± <0.01	4.73 ± 0.01	4.80 ± 0.03
Starch (%)	11.1 ± 0.10	15.6 ± 0.01	15.7 ± 0.21	15.8 ± 0.06	15.8 ± 0.10
Energy (MJ kg-1)	21.4 ± 0.09	20.9 ± 0.01	21.2 ± 0.06	21.2 ± 0.01	21.3 ± 0.01
Total phosphorous (g kg-1)	9.37 ± <0.01	8.15 ± <0.01	7.78 ± 0.02	7.80 ± 0.01	7.83 ± 0.02

Results are presented as mean (±SD).

### 2.4 Experimental design

#### 2.4.1 Fish, feeding and sample collection

Pilot experiment: At 3 mpf, fish were separated into 3 treatment groups: control high protein diet (HP), low protein (LP) and low protein diet with *C. jadinii* yeast ingredient (LPY). Each group was separated into three 3.5 L tanks with 10 zebrafish each. The initial body weight was recorded at the start of the pilot experiment. The pilot experiment lasted for four weeks and fish were fed three times per day, 08:00–09:00, 14:00–15:00 and 20:00–21:00. The daily feeding rate was 3% of the fish weight. Zebrafish were sacrificed and weighted at 14 and 28 days. The mortality was recorded throughout the pilot experiment.

Feeding trial: At 3 mpf, the fish were separated into five treatment groups: control diet (C) and 4 yeast containing diets (ICJ, ACJ, IWA, AWA). Each group was separated into two 3.5 L tanks of 20 zebrafish each. The initial body weight was recorded at the start of the feeding trial. The feeding trial lasted for four weeks and fish were fed three times per day, 08:00–09:00, 14:00–15:00 and 20:00–21:00. The daily feeding rate was 3% of the fish weight. The mortality was recorded throughout the feeding trial.

Fish were euthanized by cold ice treatment and the intestine samples were collected for proteomic analysis (6 fish per group in each analysis). Intestine samples were immediately frozen with liquid nitrogen and stored at −80 °C.

#### 2.4.2 Growth rate

The body weight gain and specific growth rate were determined at 14 and 28 days of the pilot study and the feeding trial.

Growth rate was estimated as specific growth rate (SGR) according to the equation: SGR (% day^−1^) = (lnWf-lnWi)/t × 100, where Wi is initial body weight, Wf is final body weight, and t is the period of feeding trial.

Percentage of body weight gain was calculated according to the following equation: ((Wf-Wi)/Wi) × 100 [24].

Feed conversion ratio (FCR) was calculated as: FCR = Feed DM ingested (g)/fish weight gain (g)^−1^

### 2.5 Proteomics analysis

#### 2.5.1 Protein isolation and quantification

For proteomic analysis, frozen intestinal samples from two diet groups were analyzed, namely fish group fed the diet containing autolyzed *C. jadinii* (ACJ) and the group fed the high-level protein control diet (C). The frozen intestine samples were homogenized with 200 μL of sodium dodecyl sulphate (SDS) lysis buffer (1% SDS; Sigma-Aldrich, St. Louis, MO, USA), 0.5 M triethylammonium bicarbonate buffer pH 8.5 (TEAB; Sigma Aldrich), and 1 × Protease Inhibitor cocktail (Thermo Scientific, Rockford, IL, USA) by homogenizer.

The homogenized samples were lysed by incubating at 90 °C for 30 min, and cooled on ice for 5 min. The lysed samples were centrifuged at 14,000×*g* for 20 min at 4 °C. The supernatant, containing the proteins, was transferred to a new Eppendorf tube [25]. Four volumes of ice-cold acetone were added to the protein samples and they were incubated at −20 °C overnight. The samples were centrifuged at 14,000×*g* for 10 min at 4 °C and the pellets were collected. Following which, the pellets were washed two times by centrifugation at 14,000×*g* at 4 °C for 10 min with ice-cold acetone. The pellets were dried by evaporation at room temperature to remove all the liquid and stored at −80 °C before being transported to the Department of Biological Sciences, National University of Singapore for proteomics analysis.

The pellets were first resolubilized in 5 × SDS lysis buffer. Aliquot from each sample was taken for estimating the protein concentration of the samples using a Qubit® 3.0 Fluorometer (Invitrogen, Eugene, OR, USA) and the Qubit™ Protein Assay Kit (Invitrogen) according to the manufacturer's protocol.

#### 2.5.2 S-trap digestion

**Fig. 1:**
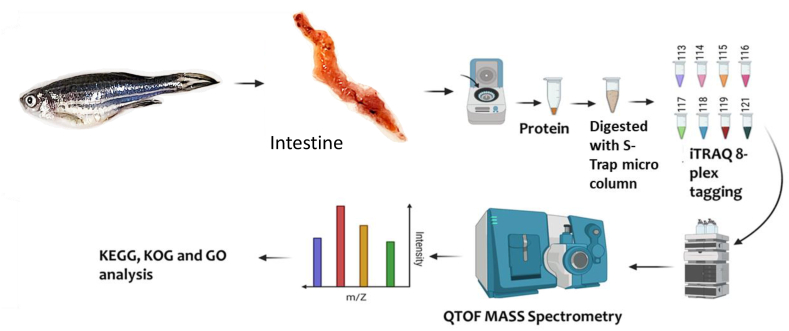
**Schematic representation of methodological approaches**. LC-MS/MS-based iTRAQ was adopted to quantify the proteins of zebrafish intestine.

In total, 100 μg of protein from intestine sample was digested using the S-Trap micro column (Protifi, Farmingdale NY, US) as per the instructions of the manufacturer. The digested peptides were quantified by Pierce quantitative colorimetric assay (Fig. 1).

#### 2.5.3 iTRAQ labelling and sample clean-up

Isobaric tags (iTRAQ) were utilized for relative quantification of proteins. One iTRAQ sample set which contained the samples from the control (C) and yeast supplemented (ACJ) diets was used to quantify the proteins. iTRAQ Reagents 8-plex kit (SCIEX, Foster City, USA) was employed to label the digested peptides. The 50 μg of dried peptide samples were re-dissolved in 30 μL of dissolution buffer (0.5 M TEAB, pH 8.5) and labelled with iTRAQ reagent. Each sample set was pooled together according to the manufacturer recommendation and sample clean-up was performed. The 1% FA was added to acidify the samples, and the samples were diluted ten-fold to obtain a 10 mL diluent (98% water, 2% ACN, and 0.05% formic acid) before the desalting process by a Sep-Pak C18 cartridge Waters (Milford, USA). The desalted eluate was lyophilized before proceeding to 2D LC-MS/MS (Fig. 1).

#### 2.5.4 2D LC-MS/MS analysis (iTRAQ)

After lyophilization of peptide samples, 2D LC-MS/MS was performed as described previously [26,27]. A high pH reversed-phase high-performance liquid chromatography unit (RP-HPLC) (1290 Infinity LC system; Agilent, Santa Clara, CA, US) equipped with a C18 column (WATERS Xbridge C18, 3.5 μm, 3.0 mm × 150 mm; Waters Milford, Massachusetts, US) was employed to perform the first-dimension separation of samples. Mobile phase A (20 mM ammonium formate in water, pH 10) and mobile phase B (20 mM ammonium formate in 80% ACN, pH 10) were used. The gradient elution profile was as follows: mobile phase B 0-0% for 10 min, 0–60% for 60 min, 60-60% for 5 min, 60–100% for 1 min, continued at 100% for 5 min and 100-0% for 1 min and subsequently for 14 min at 0%. The eluted fractions were collected in 96-well, v-bottom plates at a flow rate of 0.5 mL min^−1^. Eluted fractions were combined into 10 concatenated fractions and lyophilized.

To perform the second-dimension separation, the lyophilized samples were dissolved in 2% acetonitrile in water. For MS/MS analysis, 2 μL of each of the 10 fractions were injected independently into a ProteoCol C18P (3 μm 120 Å, 300 μm × 10 mm; Trajan) trap column and eluted on an analytical C18 analytical column (Acclaim PepMap 100C18, 3 μm particle size, 75 μm i. d. X 250 mm; Thermo Scientific) using a nanoLC 425 LC system (Eksigent) in Trap-Elute configuration. The solvents employed for the RP HPLC were mobile phase A (0.1% formic acid) and mobile phase B (0.1% formic acid in acetonitrile) to separate peptides with a flow rate of 300 nL min^−1^. The gradient elution profile was as follows: mobile phase B 5–15% for 60 min, 15–30% for 60 min, 30–90% for 3 min, continued at 90% for 20 min and 90-5% for 2 min and subsequently for 15 min at 5%. The eluted fractions were collected in 96-well plates at a flow rate of 0.5 mL min^−1^, with a flowrate of 300 nL min^−1^. The LC fractions were analyzed with a TripleTOF**®** 5600^+^ system (AB SCIEX, Framingham, Massachusetts, US) under the positive ionization mode. The conditions employed for acquiring the MS spectra were: mass range of 350–1250 m z^−1^ at high sensitivity mode with the accumulation time of 250 ms per spectrum. The 30 most abundant precursors were selected between charge range +2 to +5 per duty cycle for MS/MS analysis (accumulation time: 100 ms). In each duty cycle, 15 s dynamic exclusion was employed for MS/MS analysis in high sensitivity mode (>15,000) with rolling collision energy, and iTRAQ reagent collision adjustment settings turned-on (Fig. 1).

#### 2.5.5 Peptide and protein identification

Identification of iTRAQ-labelled proteins was performed with ProteinPilot™ 5.0.2 software (AB SCIEX); using the Paragon database search algorithm and the false discovery rate (FDR) cut-off functions [28]. The search parameters for identifying the proteins were: Sample Type: iTRAQ 8plex (Peptide Labelled); Cysteine Alkylation: MMTS; Digestion: Trypsin; Instrument: TripleTOF5600; Special Factors: None; Species: None; ID Focus: Biological Modification; Database: Zebrafish reference proteome (2022_02 release, 46687); Search Effort: Thorough; FDR Analysis: Yes. Background correction: yes; and User modified parameter files: Yes. Differential abundance of proteins was determined using paired student's *t*-test. For protein identification, 1% FDR was used as the cut-off. The differentially abundant proteins (DAPs) were selected based on the following criteria: iTRAQ ratio ≥1.3 (*P*-value <0.05) was identified as abundance-increased proteins and ≤ −1.3 (*P*-value <0.05) as abundance-decreased proteins [29] (Fig. 1).

### 2.6 Functional analysis

Eukaryotic Orthologous Groups (KOG) classification analysis was used to interpret the functions connected to the DAPs identified by comparing the protein ratios in the LP and ACJ feed groups; the classification has 4 categories [30]. The FASTA format of the protein list was obtained from the online server uniprot (https://www.uniprot.org/) and submitted into eggNOG-mapper (http://eggnogdb.embl.de/#/app/emapper), eggNOG 4.5.1 database. The obtained proteins were classified into 25 functional groups according to KOG annotation [31]. To reveal the Gene Ontology (GO) and Kyoto Encyclopedia of Genes and Genomes (KEGG) pathway linked to DAPs, ShinyGO 0.76.3 was used [32].

### 2.7 Statistics

Data of percentage of body weight, SGR and FCR were represented as means values with standard deviation. The outliers were identified by Tukey JW, 1997 by categorizing ‘outside’ values, subsequently the outliers were removed from further analysis. Post-ANOVA multiple comparisons between the mean of each group with mean of every other group were performed by Dunn's test (adjusted *P*-value) was <0.05 with 95% confidence interval. The statistical analyses were performed by using GraphPad Prism version 9.5.0 for Windows (GraphPad Software, La Jolla, CA, USA; www.graphpad.com). Growth performance parameters are presented as diet means ± standard error of the mean.

## 3 Results

### 3.1 Fish growth and survival

In the pilot study at 14 days, the high protein diet group (HP) had a higher body weight gain (*P*-value = 0.1) and specific growth rate (SGR; *P*-value = 0.1) than low protein diet group (LP and LPY), but the differences was not statistically significant (*P* > 0.05). At 28 days, the HP diet group had a significantly higher body weight gain (*P*-value = 0.04) and SGR (*P*-value = 0.04) than the LP diet group but not with the LPY diet group (Supplementary File S2). No fish died during the pilot study.

In the feeding trial, there were no significant differences (*P* > 0.05) in body weight gain, SGR and feed conversion ratio (FCR) observed between fish fed with five diets at 14 and 28 days (Table 3). Two fish fed the C diet (one in each tank) died before day 14 of the feeding trial (5% mortality in Diet LP). No fish fed the other four diets died during the feeding trial.

**Table 3:** Growth performance of zebrafish fed control and yeast diets for 14 and 28 days.

Growth parameters Diet SEM											
	C	ICJ	ACJ	IWA	AWA	C	ICJ	ACJ	IWA	AWA	*P*-value
**Initial weight (g)**											
0 Day	1.73	1.93	1.8	1.76	1.81	0.07	0.05	0.08	0.05	0.01	
**Final weight (g)**											
14 Days	2.92	3.43	3.26	3.08	2.83	0.25	0.18	0.08	0.23	0.33	
28 Days	3.72	4.71	3.5	4.27	4.52	0.92	0.12	0.4	0.31	0.07	
**Weight gain %**											
14 Days	70.1	77.6	83.1	75.3	56.5	21.1	4.73	3.78	8.61	19.1	0.9
28 Days	118	144	100	143	149	61.5	0.36	31.8	11.2	4.97	0.9
**SGR (%)**											
14 Days	3.74	4.11	4.32	4.00	3.14	0.88	0.19	0.15	0.35	0.88	0.9
28 Days	2.63	3.18	2.44	3.16	3.26	1.04	0.01	0.57	0.16	0.07	0.9
**FCR**											
14 Days	1.97	1.63	1.52	1.70	2.52	0.59	0.1	0.07	0.19	0.85	0.8
28 Days	3.80	2.43	3.94	2.44	2.16	1.76	0.05	1.20	0.11	0.09	0.6

SEM, standard error mean; SGR, standard growth rate and FCR, feed conversion ratio.

### 3.2 Proteins overview

We quantified 3460 proteins from the intestinal samples of fish fed the control and ACJ diets, of which, 2450 proteins were identified using a false discovery rate (FDR) of 1% and with at least one peptide (confidence interval ≥95%). Among the identified proteins, 277 proteins had a fold change ≥1.3 or ≤ −1.3 and *P*-value <0.05. These 277 differentially abundant proteins (DAPs) were assumed to be affected by the ACJ diet. Among these DAPs, 124 proteins were abundance-increased (higher abundance proteins; HAP) and 153 were of abundance-decreased (lower abundance proteins; LAP) in fish that were fed the ACJ diet (Supplementary File S3).

### 3.3 Annotation and functional classification

**Fig. 2:**
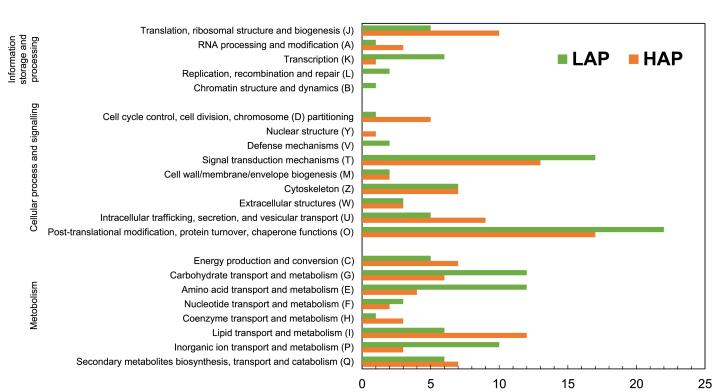
EuKaryotic Orthologous Groups (KOG) functional classification of 124 high abundant proteins and 153 low abundant proteins from the intestines of zebrafish fed with ACJ diet. LAP: lower abundance proteins; HAP: higher abundance proteins.

**Fig. 3:**
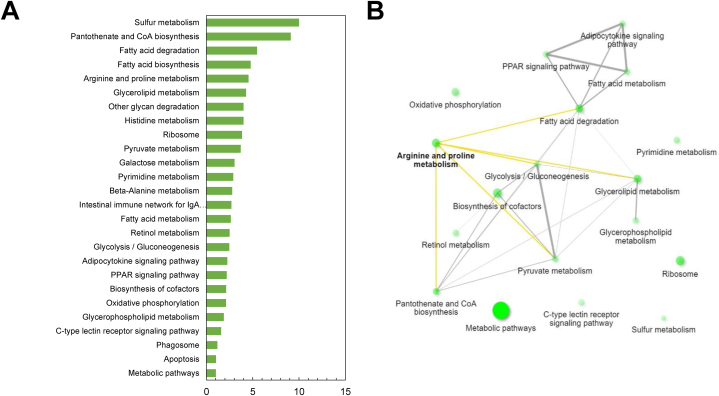
List of KEGG pathways (Panel A) and associated protein network (Panel B) of high abundant proteins from the intestines of zebrafish fed with ACJ diet. Pathways listed at least two proteins involved. Panel A: X-axis labels display the percentage of proteins linked to the GO terms shown in the Y-axis.

**Fig. 4:**
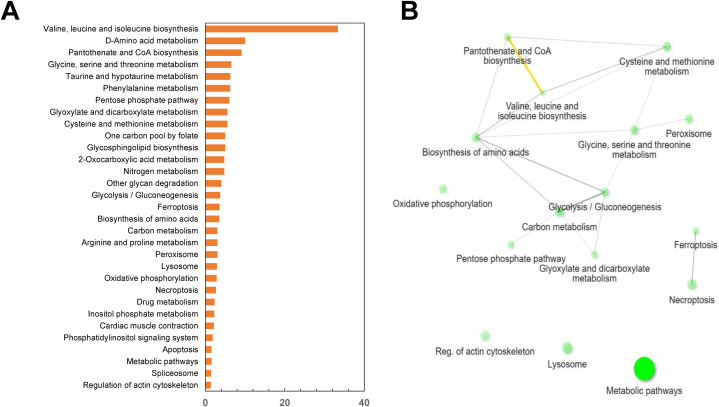
List of KEGG pathways (Panel A) and associated protein network (Panel B) of low abundant proteins from the intestines of zebrafish fed with ACJ diet. Panel A: X-axis labels display the percentage of proteins linked to the GO terms shown in the Y-axis.

The quantified proteins by iTRAQ were subjected to EuKaryotic Orthologous Groups (KOG) and Kyoto Encyclopedia of Genes and Genomes (KEGG) pathway analysis. The KOG analysis demonstrated distribution into three functional categories and further into 22 groups (Fig. 2). In both HAPs and LAPs, “posttranslational modification, protein turnover, chaperones functions” was the most represented group, followed by “signal transduction mechanisms”. The functional groups “lipid transport and metabolism”, and “translation, ribosomal structure and biogenesis” were highly represented in HAPs. On the other hand, “amino acid transport and metabolism”, and “carbohydrate transport and metabolism” were highly represented in LAPs. Regarding HAPs, 124 proteins were annotated and classified into metabolism, cellular processes and signaling and information storage and processing categories (38.3%, 49.6% and 12.1%, respectively). For the LAPs, 153 annotated proteins were distributed amongst these same categories at 43%, 45% and 12, respectively (Fig. 2). KEGG analysis revealed that the HAPs belonged to several pathways related to immune functions, including ribosome (40 S ribosomal protein S4, 40 S ribosomal protein S7, 40 S ribosomal protein SA, 60 S ribosomal protein L28 & 60 S ribosomal protein L30), arginine and proline metabolism (creatine kinase, aldehyde dehydrogenase), PPAR signaling pathway (Acyl-CoA synthetase long chain family member 4a & carnitine O-palmitoyltransferase), C-type lectin receptor signaling pathway (ADP-ribosylation factor 1 & RAS-related 2) and phagosome (tubulin alpha chain & ATPase H+ transporting V0 subunit e1). The protein network associated with HAPs are shown (Fig. 3 & Supplementary File S3). The LAPs were connected to pathways related to inflammatory functions such as necroptosis (signal transducer and activator of transcription, peptidyl-prolyl *cis*-trans isomerase & calpain-1 catalytic subunit), ferroptosis (ferritin) and apoptosis (cathepsin S & cathepsin L). The protein networks connected with LAPs are shown (Fig. 4 & Supplementary File S3). Selected pathways and their associated proteins identified from both the high and low abundant protein list are summarised in supplementary file S4.

**Fig. 5:**
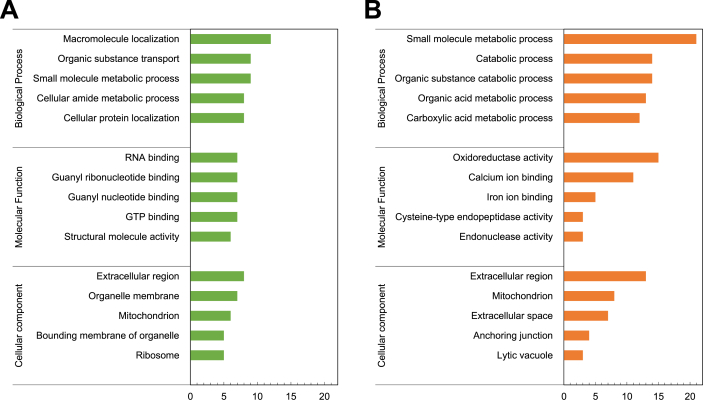
**Significantly enriched Gene Ontology (GO) terms of the high abundant and low abundant proteins from intestines of zebrafish fed with ACJ diet.** Top five GO terms based on the protein numbers are shown. P < 0.05. X-axis labels display the number of proteins linked to the GO terms shown in the Y-axis. Panel A: high abundant proteins; Panel B: low abundant proteins.

The GO enrichment analysis of HAPs showed that macromolecule localization, small molecule metabolic process and organic substance transport were connected to the biological processes category. The GO term GTP binding, guanyl nucleotide binding and guanyl ribonucleotide binding were related to the molecular function category and extracellular region, organelle membrane and mitochondrion under cellular component were highly enriched in HAPs (Fig. 5 & Supplementary File S5).

For GO enrichment analysis of LAPs, biological processes related to small molecule metabolic process, organic substance catabolic process and catabolic process, were connected to the molecular function category. Oxidoreductase, calcium ion binding and iron ion binding and from cellular component category, the GO term extracellular region, mitochondrion extracellular space were the most representative terms in LAPs (Fig. 5 & Supplementary File S5).

## 4 Discussion

This study used zebrafish as a model organism to investigate the influence of a yeast-based feeds on fish physiological processes. The well-annotated proteome of zebrafish and the research tools available for its detailed investigation have enabled insights into the underlying molecular mechanisms of immune processes that are relevant for a range of aquaculture species. Our study showed that the inclusion of *C. jadinii* in the experimental diet modulated the intestinal proteome of zebrafish. The modulation was in the intestinal proteomic pathways related to cell populations and the innate immune response.

In the present study, we used a yeast-based feed that has been shown to alleviate diet-based inflammation in Atlantic salmon [4,20]. We found that the yeast-based diet did not have adverse effects on zebrafish nor were zebrafish adversely affected by another yeast strain that had also been tested in Atlantic salmon diets. Agboola and co-workers (2022) described a modulation of innate immunity through taurine and hypotaurine pathways. Detailed proteomic analysis of the intestines from zebrafish fed the ACJ diet showed that many of the 124 HAPs were involved in pathways related to the innate immune system.

### 4.1 Arginine and proline metabolism

KEGG identified HAPs related to arginine and proline metabolism that were linked to other pathways containing HAPs including glycerolipid metabolism, glycolysis/gluconeogenesis, fatty acid degradation, pyruvate metabolism and pantothenate and CoA biosynthesis. Arginine and proline metabolism is at the base of a fundamental division of macrophage biology [33,34]. It has been shown in mammals and fish that macrophages adopt polarization states that exert very different effects [35]. This functional difference has been described as a spectrum of activation states with M1 and M2 macrophages at opposite ends of the spectrum. Inflammatory M1 macrophages metabolize arginine to produce anti-microbial nitric oxide while anti-inflammatory M2 macrophages use arginine to produce proline and polyamines for cell proliferation and tissue regeneration. In demonstrating M1/M2 polarization in fish macrophage, Wentzel and co-workers speculated on the importance of immunostimulants for aquaculture, particularly the ability to steer innate immune responses to achieve sustainable improvements in fish health. The finding in the present study that feeding a yeast-based diet to a laboratory fish species modulated arginine and proline metabolism in the intestine should be investigated further to determine the activation state of macrophages in the intestines of fish fed this diet and its functional significance in relation to disease and stress resistance.

### 4.2 Recognition of *C. jadinii* through phagosome and C-type lectin receptors

KEGG analysis also identified other immune-related pathways among HAPs. The analyses indicated that phagosome and C-lectin receptor signaling are closely associated with high abundant proteins in fish fed the ACJ diet. As with mammals, fish have diverse phagocyte populations including neutrophils, dendritic cells-like (DC-like), and macrophages, which are involved in the recognition and elimination of foreign bodies such as bacteria and other pathogens. Phagocytosis is facilitated by hydrophobic or sugar/lectin interactions [[36], [37], [38]]. The C-type lectin receptors (CLR) facilitate the early recognition of microbes by antigen presenting cells (APCs) such as macrophages, or DCs, to initiate an immune response [39,40] and eventually eliminate pathogens. The CLRs have anti-viral [41], anti-fungal [42] and anti-bacterial properties [43]. CLRs bind to pathogen structures known as Microbe- or Pathogen-associated molecular patterns (MAMPs/PAMPs) to help in maturation of APCs [44,45]. In addition, the CLRs take part in processes such as cell adhesion, tissue integration and remodelling, platelet activation, complement activation, pathogen recognition, endocytosis, and phagocytosis [46]. The identification of phagosome and C-type lectin receptor pathways adds support to the notion that intestinal macrophage populations are modulated in fish fed the ACJ diet.

### 4.3 PPAR signaling pathway

Peroxisome proliferator-activated receptors (PPARs) are a family of nuclear hormone receptors that regulate immune and inflammatory responses. The activation of these nuclear receptors during an inflammatory response modulates the pro-inflammatory response, preventing it from being excessively activated [47]. In fish, the PPAR-δ and -α homologues have been cloned from the orange-spotted grouper, *Epinephelus coioides* (EcPPAR-δ and EcPPARα) [48,49]. Investigation of expression of these homologues in relation to viral infection Singapore grouper iridovirus (SGIV) in grouper spleen cells found that overexpression of EcPPAR-δ reduced the SGIV replication while overexpression of EcPPARα increased replication of SGIV. These studies concluded that EcPPAR-δ had a positive role in interferon signaling pathway regulation, antiviral response and anti-inflammatory effect on the inflammatory reaction and inhibition of SGIV replication [48] while increased SGIV replication following over expression of EcPPARα may be due to the downregulation of inflammatory response and interferon [49]. Interestingly, groupers challenged with the bacterium *Vibrio alginolyticus* showed a sharp increase of EcPPAR-δ transcript in immune tissues and it was speculated that EcPPAR-δ was a negative regulator of pro-inflammatory cytokines that played an important role in the immune defense against vibrio-induced inflammation in grouper [50]. The highly abundant presence of proteins from the PPAR signaling pathway in zebrafish fed the ACJ diet suggests that the yeast containing diet may modulate pro-inflammatory responses.

### 4.4 The diet containing *C. jadinii* enhances the immune boosting effect on zebrafish gut

In addition to the immune related pathways, ribosome pathway was also found to be associated with HAPs. The ribosome pathway includes ribosomal proteins, namely 40 S ribosomal proteins S4, SA, S7 and 60 S ribosomal proteins, L30 and L28. Changes in levels of ribosomal proteins are associated with cell proliferation and apoptosis [51].

An interesting protein in the HAPs was the soluble N-ethylmaleimide-sensitive factor (NSF) attachment protein (SNAP). SNAP is a key element of vesicle trafficking machinery that controls cell fate through either pro-death or pro-survival signaling. This protein enhances the resistance of cells to cytotoxic stimuli and facilitates the survival of epithelial cells by controlling Golgi biogenesis and Bcl-2 expression [52,53]. This vesicle-trafficking protein is also essential during phagocytosis facilitating endoplasmic reticulum fusion with plasma membrane [54,55].

A further HAP was the multifunctional enzyme, glutathione S-transferase (GST) which plays a key role in cellular defense mechanisms in most organisms against toxic compounds. GSTs are typically involved in the innate immunity and detoxification of harmful xenobiotics by using one of the thiol group of glutathione (GSH) conjugation [56].

Thus, many HAPs were represented in innate immune pathway indicating that the ACJ based yeast diet had an immune boosting effect on zebrafish gut. This finding is similar to the health-beneficial effects reported in the previous study on Atlantic salmon [4]. It should also be noted that the identification of immune enhancing effect of the feed across two distinct orders, Cypriniformes (zebrafish) and Salmoniformes (Atlantic Salmon) suggests that the feed could have similar effects in other aquaculture species.

### 4.5 The yeast suppresses inflammation related proteins

Apoptosis pathways were represented in both HAP and LAP. There were 2 proteins, ADP-ribosylation factor 1 and tubulin alpha chain present in the HAP, while there were three proteins, calpain 2, (m/II) large subunit a, and cathepsin B and L present in the LAP. Calpain is known to increase inflammation by degrading IκB, the endogenous inhibitor of nuclear factor-kappa B (NF-κB). Activated NF-κB, a master transcriptional regulator in turn regulates numerous inflammatory mediators, such as tumor necrosis factor alpha (TNF-α), interleukin 1 beta (IL-1β) and cyclooxygenase-2 [57]. Conversely, calpain inhibitor is shown to have a protective role in the inflammation-associated disease [58,59]. Also, it was noteworthy to find cathepsin B and L in LAP as these proteins are associated with intracellular protein degradation and enhanced expression is seen in inflammatory diseases. It has been reported that cathepsin B inhibition prevents appearance of multinucleated cells, which is an early indicator of microtubule stabilizing agents (MSAs)- induced cell death pointing to cathepsin's role in the cell death pathway [60]. Lower abundance of cathepsins in fish fed the ACJ based diet could thus suggest the beneficial effect of yeasts in preventing mucosal damage and cell death [61].

The analysis of LAPs identified in the intestine of fish fed the yeast-based diet found pathways related to necroptosis and ferroptosis, which are usually associated with increased inflammation [62,63]. These identified LAPs included signal transducer and activator of transcription, calpain-1 catalytic subunit, peptidyl-prolyl *cis*-trans isomerase and ferritin, respectively, which adds further evidence for the suppression of inflammation in response to feeds formulated with the autolyzed yeast extract, ACJ. The identification of these pathways by LAP adds weight to the notion that ACJ based diets contribute to the suppression of an inflammatory response.

### 4.6 Macrophage migration inhibitory factor

A further LAP in the intestine of zebrafish fed the ACJ diet was macrophage migration inhibitory factor (MIF). MIF can stimulates the expression of proinflammatory cytokines, such as tumor necrosis factor alpha (TNF-α), interleukin 1 beta (IL-1β), interleukin 6 (IL-6), interleukin 8 (IL-8), and interferon-gamma (IFN-γ) through the natural receptor cluster of differentiation 74 (CD74) that enhance the inflammatory reaction in humans [64]. High-level gastrointestinal expression of MIF in human is associated with occurrence of several diseases such as gastritis, gastric malignancy, gastritis ulcer, colon cancer and ulcerative colitis [65]. Increased level of MIF was observed during *Helicobacter pylori* infection and was substantially reduced after eradication by a first-line eradication regimen [66,67]. MIF promotes the proinflammatory function of macrophage in mice with p53 inhibition [68]. Another study explained that MIF was essential for Japanese sea bass during *V. harveyi* infections but found that higher expression of MIF was harmful in acute infection [69]. In addition, Wu, Yang [70] revealed that MIF in grass carp enhanced the secretion of inflammatory factors IL-1β, TNF-α and IL-6 from leukocytes. The low abundance of MIF argues for a modulation of macrophage populations in the intestines of fish fed the ACJ diet.

It is well established that certain soybean-soy saponin combinations induced distal intestine (DI) inflammation in Atlantic salmon [71,72]. The transcriptional changes associated with inflammation have been linked to disturbances in transport mechanisms as well as drug metabolism and taurine and hypotaurine metabolism and steroid biosynthesis in the DI of the fish [73]. The presence of LAPs from these pathways in the intestine of zebrafish fed ACJ diets is consistent with the abilities of ACJ diets to alleviate diet-based inflammation typically caused by plant-based ingredients in Atlantic salmon [4].

### 4.7 Novel ingredients and the importance of immunoregulation in the fish intestine

Lee, Yamamoto [74] proposed that the use of orally administered compounds is involved in immune homeostasis of the fish gut, coordinating the physiological response of the organism.

The intestine, is a mucosa associated lymphoid tissue (MALT), which contains cells (e.g., lymphocytes, phagocytes, APCs) and humoral components (cytokines and effector molecules) that modulate local responses in the intestine and systemic responses in other MALTs such as gills and skin and immunological organs such as head kidney and spleen [75,76]. Thus, the intestine can be considered a primary target to regulate the host response when novel yeast-based products are used, since these compounds contain MAMPs (e.g., glucans, mannans and nucleic acids) that can prime the immune system by the activation of Pattern Recognition Receptors (PRRs) such as CLRs and Toll-like receptors (among others) [76,77].

In fish, such as salmonids and zebrafish, it has been reported that plant-based ingredients may induce an inflammatory response in the intestine [78,79]. For example, soybean meal, which also contains antinutritional factors, affects fish growth and modulates an exacerbated immunological response, which is harmful to the host [80]. Therefore, being able to use alternative and sustainable protein sources that do not negatively impact fish health and welfare is an important challenge to be addressed for global aquaculture. Our study contributes to the identification of alternative protein sources since we have detected that the inclusion of *C. jadinii* (ACJ) in novel feeds has modulated metabolic pathways associated with the activation and balance between the M1/M2 macrophage populations. (e.g., C-type lectin receptors, arginine and proline metabolism, PPAR signaling pathway). In addition, ACJ was also found to suppress the production of proteins involved in cellular processes related to inflammation (e.g., ferroptosis, apoptosis, necroptosis, migration inhibitory factors). These findings suggest a role for ACJ in activating and/or balancing the immune response, which could be relevant to alleviate stressful conditions related to suboptimal nutrition in farmed fish.

## 5 Conclusion

The detailed analysis of innate immune response pathways in the intestinal proteome of zebrafish fed a diet containing *C. jadinii* yeast revealed several links to macrophages and antigen-presenting cells and pathways relating to anti-inflammatory and tissue repair processes. These findings suggest that 10% inclusion level of *C. jadinii* mobilized innate immune responses in the laboratory fish species investigated. The well annotated proteome of zebrafish enables insight into the health-beneficial effects of the yeast species *C. jadinii*, which are relevant for aquaculture species. Further studies should address the functional significance of this modulation for disease resistance and the sustainable improvement of fish health.

## Funding

This research was funded by Foods of Norway, a Centre for Research-based Innovation at the Norwegian University of Life Sciences (NMBU), funded by The Research Council of Norway (SFI/NFR; 237841/O30). CRISPR/Cas9 editing (Research Council of Norway project no. 283541); International Travel Grand, NMBU (for K.P.).

## Data availability

The mass spectrometry proteomics data have been deposited to the ProteomeXchange Consortium via the PRIDE [1] partner repository with the dataset identifier PXD040431.

Account details: Username: reviewer_pxd040431@ebi.ac.uk.

Password: Eop5QpZA.

## CRediT authorship contribution statement

**Kathiresan Purushothaman:** Writing – original draft, Visualization, Validation, Software, Methodology, Investigation, Formal analysis, Data curation, Conceptualization. **Alexander D. Crawford:** Writing – review & editing, Validation, Methodology, Investigation. **Sérgio D.C. Rocha:** Writing – review & editing, Visualization, Methodology, Investigation, Formal analysis, Data curation, Conceptualization. **Aleksandar B. Göksu:** Writing – review & editing, Validation, Methodology, Formal analysis, Data curation. **Byron Morales Lange:** Writing – review & editing, Validation, Methodology, Investigation, Formal analysis, Conceptualization. **Liv Torunn Mydland:** Writing – review & editing, Validation, Investigation, Funding acquisition, Formal analysis, Data curation, Conceptualization. **Shubha Vij:** Writing – review & editing, Validation, Investigation, Data curation. **Lin Qingsong:** Writing – review & editing, Validation, Methodology, Investigation, Formal analysis, Data curation. **Margareth Øverland:** Writing – review & editing, Supervision, Resources, Project administration, Investigation, Funding acquisition, Formal analysis, Data curation, Conceptualization. **Charles McL. Press:** Writing – review & editing, Supervision, Resources, Project administration, Methodology, Investigation, Funding acquisition, Data curation, Conceptualization.

## Declaration of competing interest

The authors declare no competing interests.
